# Supplementary material for: Structure of voltage-modulated sodium-selective NALCN-FAM155A channel complex
Source: Nat Commun. 2020 Dec 3;11:6199. doi: 10.1038/s41467-020-20002-9 (PMC7712781; doi:10.1038/s41467-020-20002-9)
Supplement: Supplementary file 1 — Supplementary Information [file 41467_2020_20002_MOESM1_ESM.pdf]

Supplementary Information for

Structure of voltage-modulated sodium-selective NALCN-FAM155A channel complex

This PDF file contains:

Supplementary Figs. 1-10

Supplementary Table 1

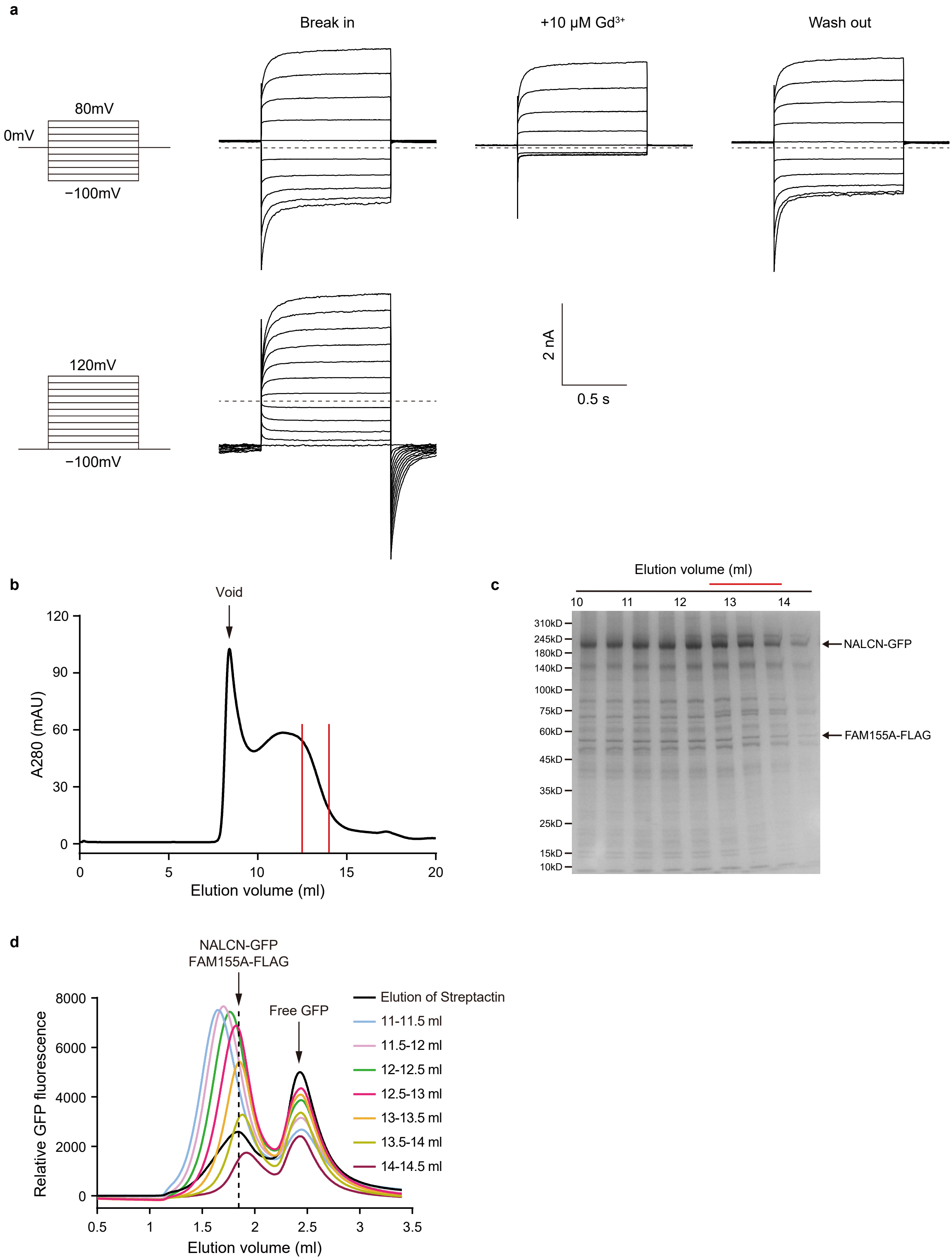

Supplementary Fig. 1 | Electrophysiology and biochemistry characterization of NALCN channel complex. a, Representative current traces of HEK293 cells expressing C-terminal GFP-tagged rNALCN, C-terminal FLAG-tagged mFAM155A, N-terminal mScarlet-tagged mUNC80 and N-terminal HA-tagged mUNC80 in the symmetric sodium solutions. 10 µM $GdCl_3$ was added to the bath solution to block the inward current. Dashed lines indicate the position of 0 nA. **b**, Size-exclusion chromatography of NALCN-FAM155A complex on a Superose 6 increase column. The fractions between the red vertical lines were pooled and concentrated for cryo-EM sample preparation. **c**, SDS-PAGE of fractions from size-exclusion chromatography. The bands corresponding to NALCN and FAM155A protein were indicated. The fractions indicated with red line were used for cryo-EM sample preparation. The experiments were repeated twice with similar results. The original gel was provided as a Source Data file. **d**, Fluorescence-detection size-exclusion chromatography (FSEC) of purified NALCN-FAM155A complex from Superose 6 columns. The peak position of elution from Streptactin was indicated as dashed line. Only fractions of Superose 6 with similar peak position compared to elution of Streptactin were pooled and used for cryo-EM sample preparation.

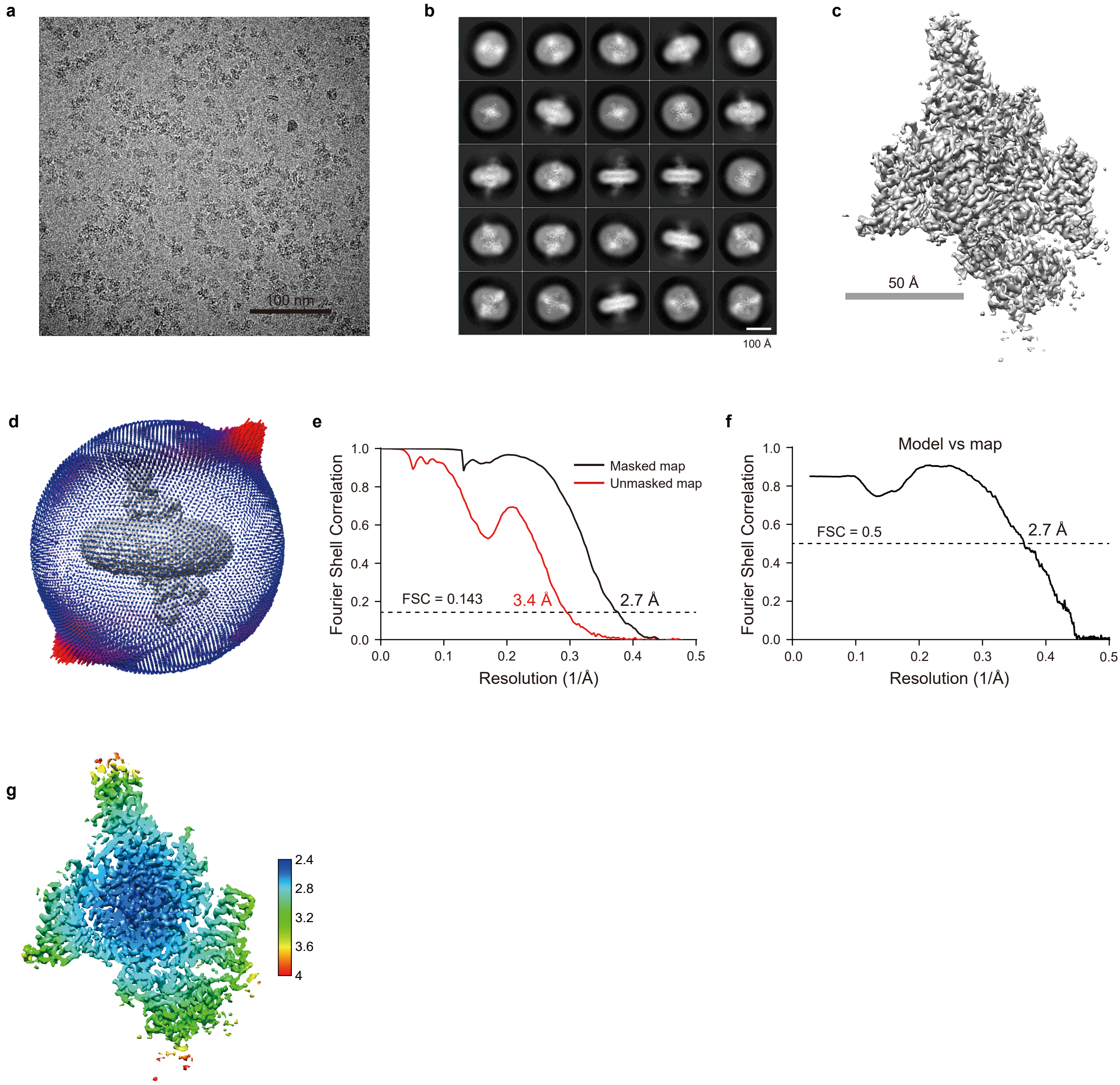

Supplementary Fig. 2 | Cryo-EM analysis of NALCN-FAM155A complex. a, One representative raw micrograph of NALCN-FAM155A complex sample out of 5,470 micrographs collected. **b**, Representative 2D class average of NALCN-FAM155A particles. Scale bar, 10 nm. **c**, Cryo-EM map of NALCN-FAM155A complex. **d**, The angular distribution of final reconstruction. **e**, Gold standard FSC curves of NALCN-FAM155A unmasked and masked EM map. Resolution estimations are based on the criterion of FSC 0.143 cutoff. **f**, FSC curve of the refined model versus EM map. **g**, Local resolution map of NALCN-FAM155A complex calculated using Relion 3.1.

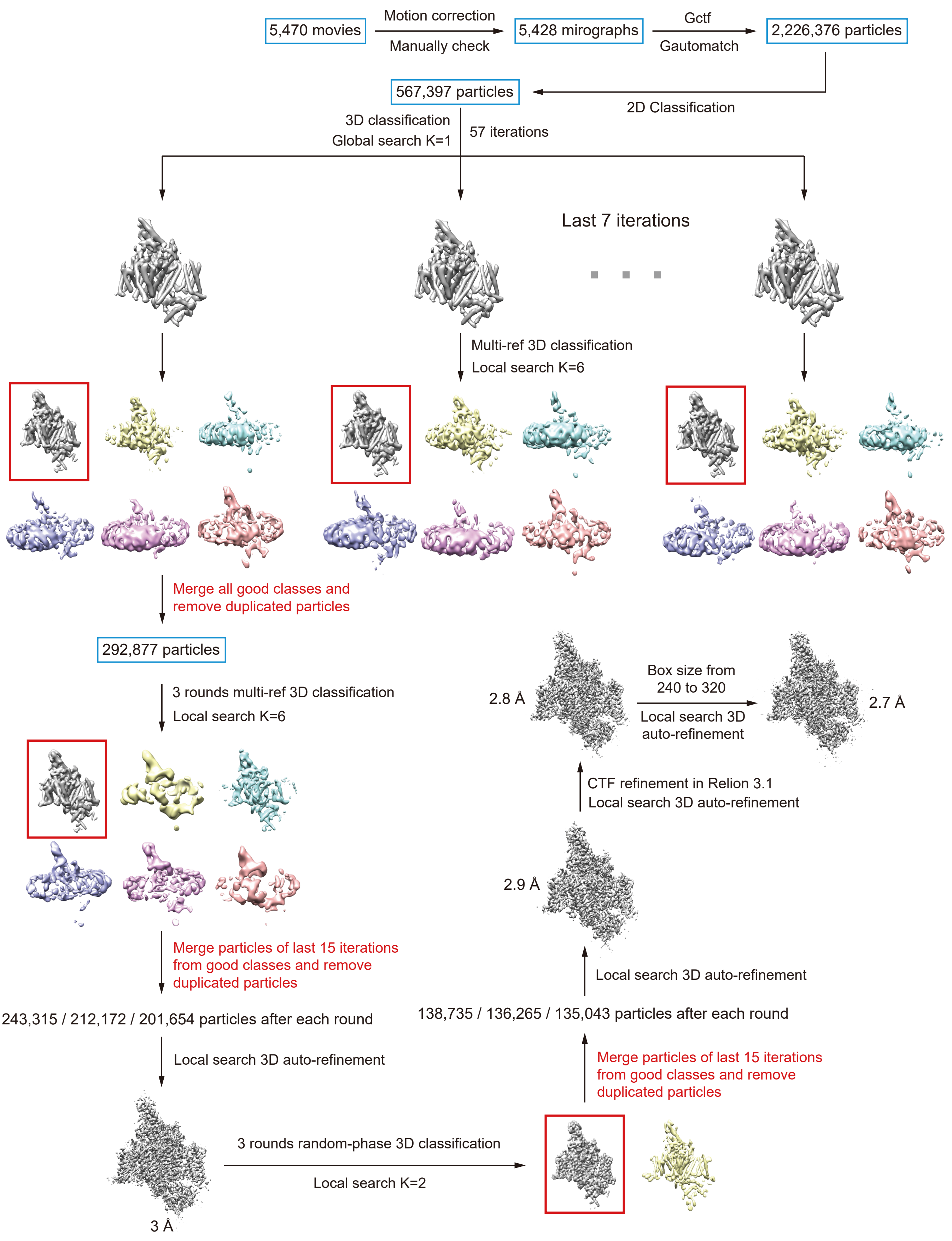

Supplementary Fig. 3 | Workflow for cryo-EM data processing of NALCN-FAM155A complex. For details, see 'Image processing' in Methods section.

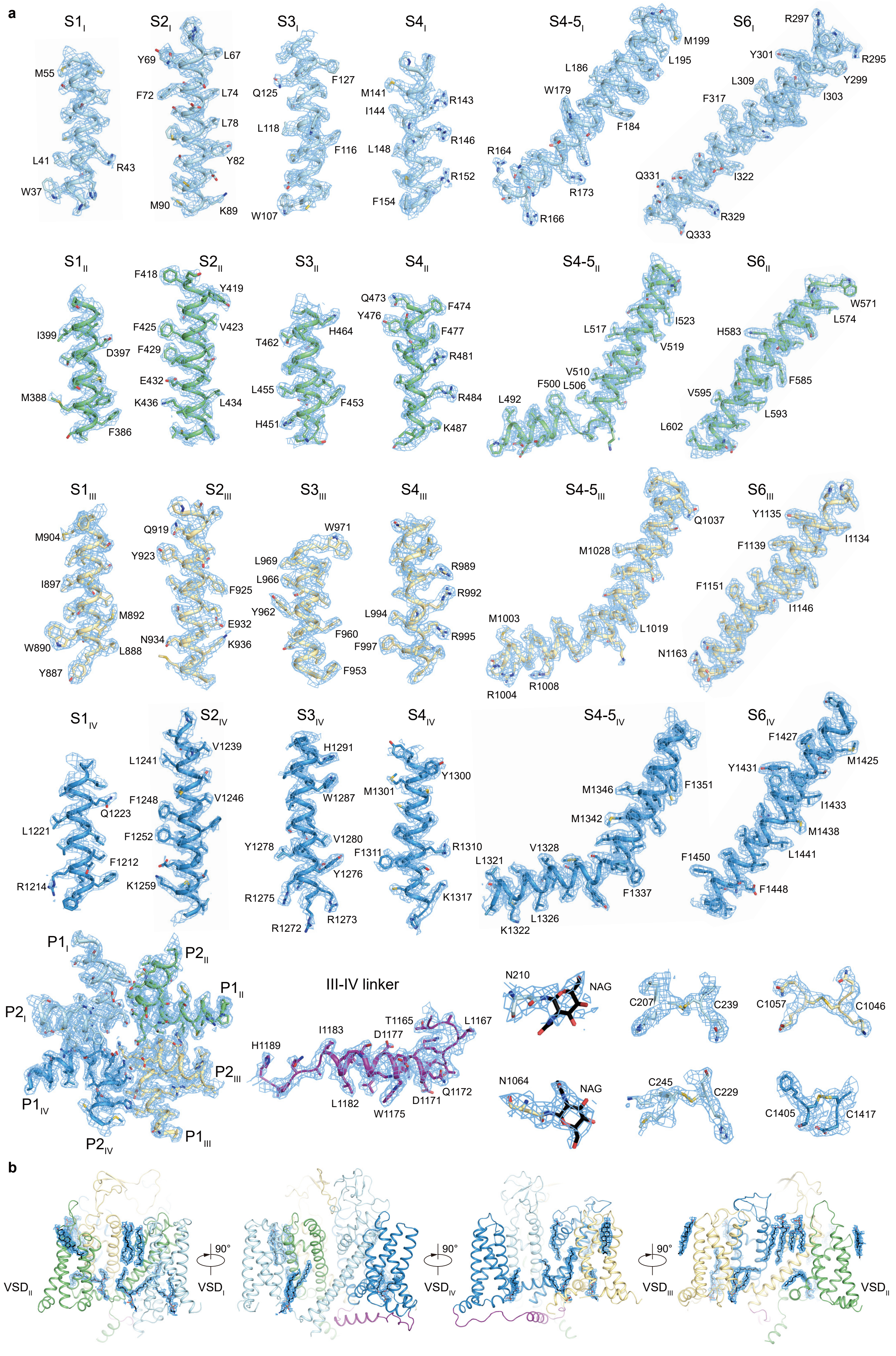

Supplementary Fig. 4 | Representative EM maps for segments in NALCN subunit. a, EM densities for S1-S6 transmembrane segments, pore domain, III-IV linker, sugar moiety and disulfide bonds in NALCN subunit, shown as blue mesh, are contoured at 3.5 σ. **b**, EM densities of putative lipids around NALCN protein, shown as blue mesh, are contoured at 2.5 σ. For clarity, intracellular CIH and CTD domains of NALCN subunit and FAM155A subunit are omitted.

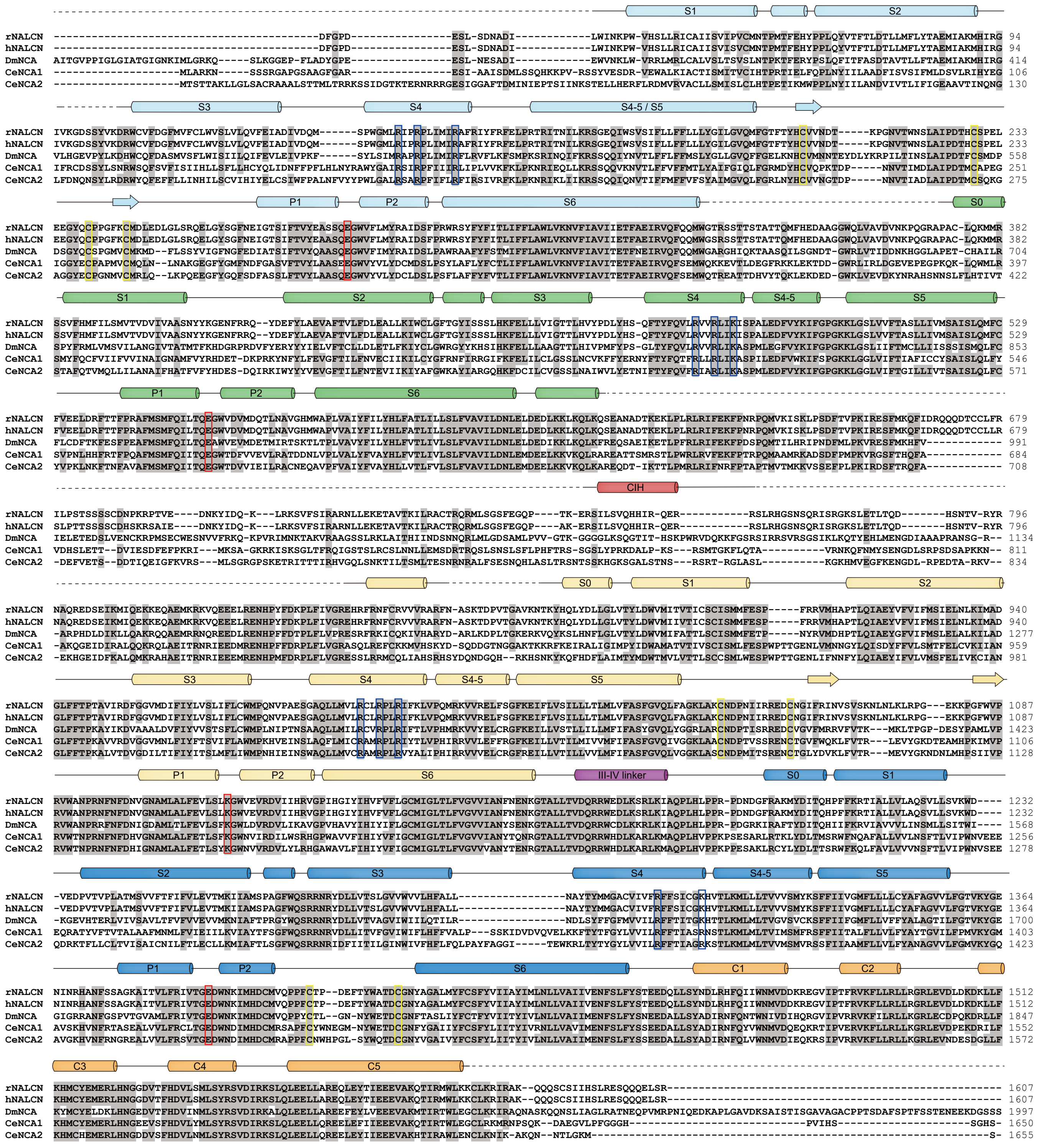

Supplementary Fig. 5 | Sequence alignment of NALCN subunit. The sequences of rat NALCN, human NALCN, fruit fly NCA, nematode NCA1 and NCA2 were aligned. Conserved residues are shaded in grey. Residues forming disulfide bonds, the predicted selectivity filter "EEKE", and the positive residues on S4 segments are indicated by yellow, red, and blue boxes, respectively. Secondary structural elements are indicated as follows: arrows, β-sheets; cylinders, α-helices; lines, loops. Disordered regions are shown as dashed lines.

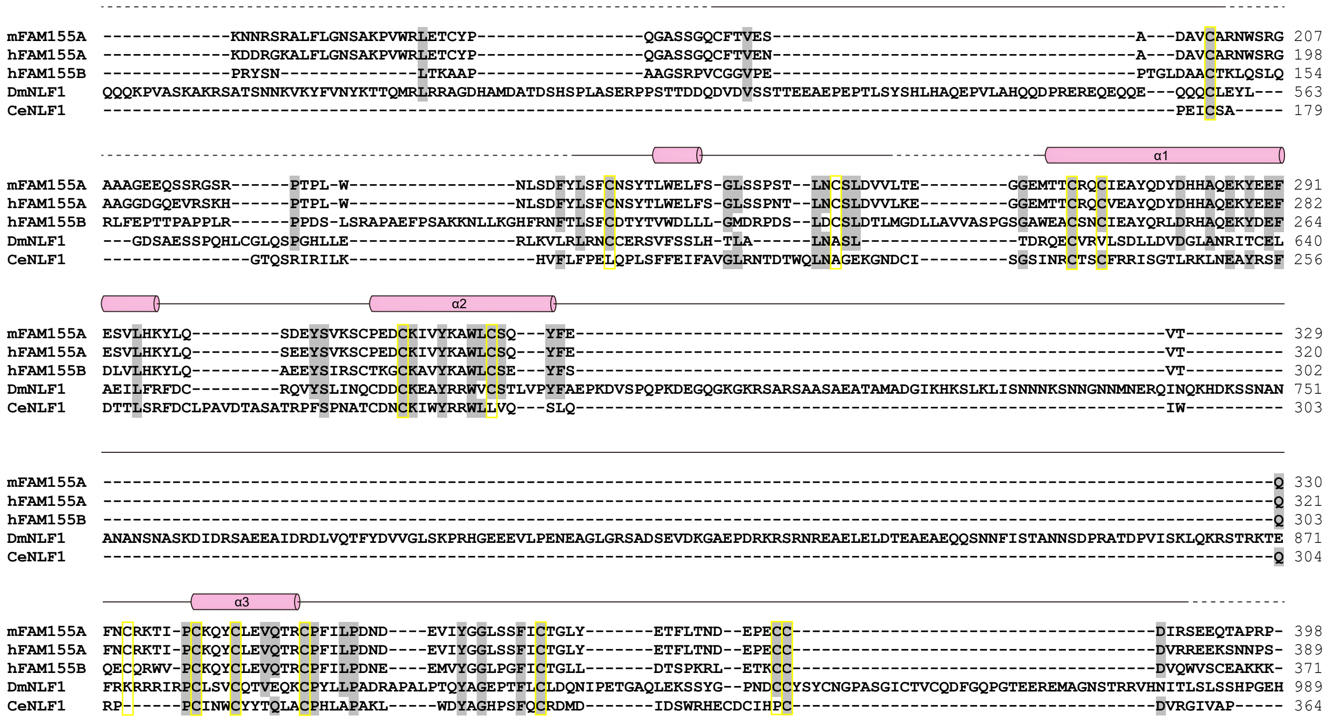

Supplementary Fig. 6 | Sequence alignment of FAM155A subunit. The sequences of mouse FAM155A, human FAM155A, human FAM155B, fruit fly NLF1 and nematode NLF1 were aligned. Conserved residues are shaded in grey. Residues forming disulfide bonds are indicated by yellow boxes. Secondary structural elements are indicated as follows: cylinders, α-helices; lines, loops. Disordered regions are shown as dashed lines.

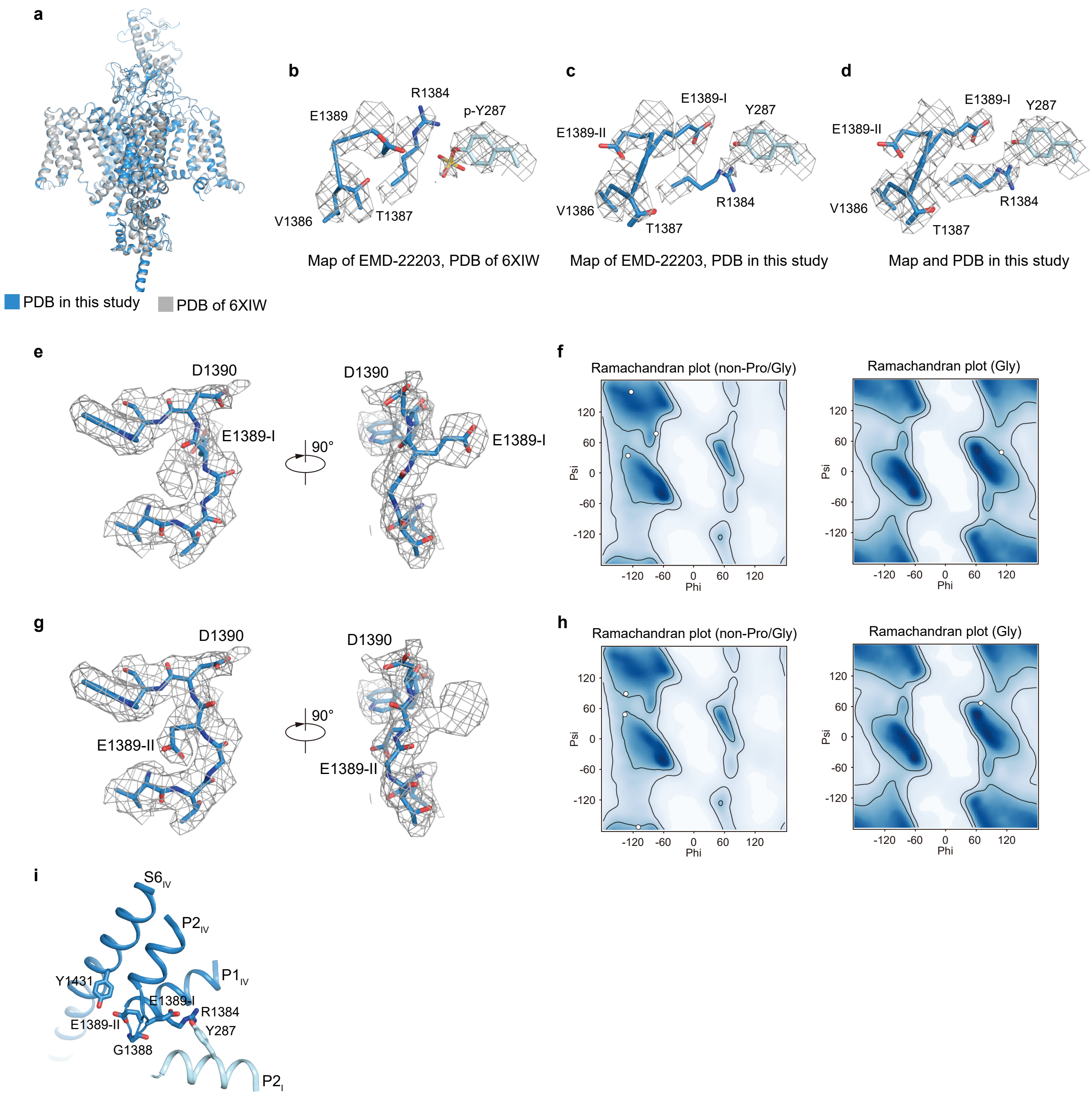

Supplementary Fig. 7 | Structure of the selectivity filter of NALCN. **a**, Overall structural comparison of NALCN-FLAM155A complex between this study (blue) and 6XIW (grey). **b**, Electron density of the selectivity filter (grey) of EMD-22203 superposed with atomic model of PDB 6XIW, is contoured at 4 σ. **c**, Electron density of the selectivity filter (grey) of EMD-22203 superposed with atomic model in this study, is contoured at 4 σ. **d**, Electron density of the selectivity filter (grey) in this study superposed with atomic model in this study, is contoured at 4 σ. **e**-**h**, Electron densities and Ramachandran plots of the selectivity filter of DIV (residues 1386-1391, shown as sticks) in E1389-I conformation (**e**, **f**) and E1389-II conformation (**g**, **h**). Electron densities are shown as grey mesh, contoured at 3 σ. **i**, Detailed interactions involving two conformers of E1389, E1389-I and E1389-II.

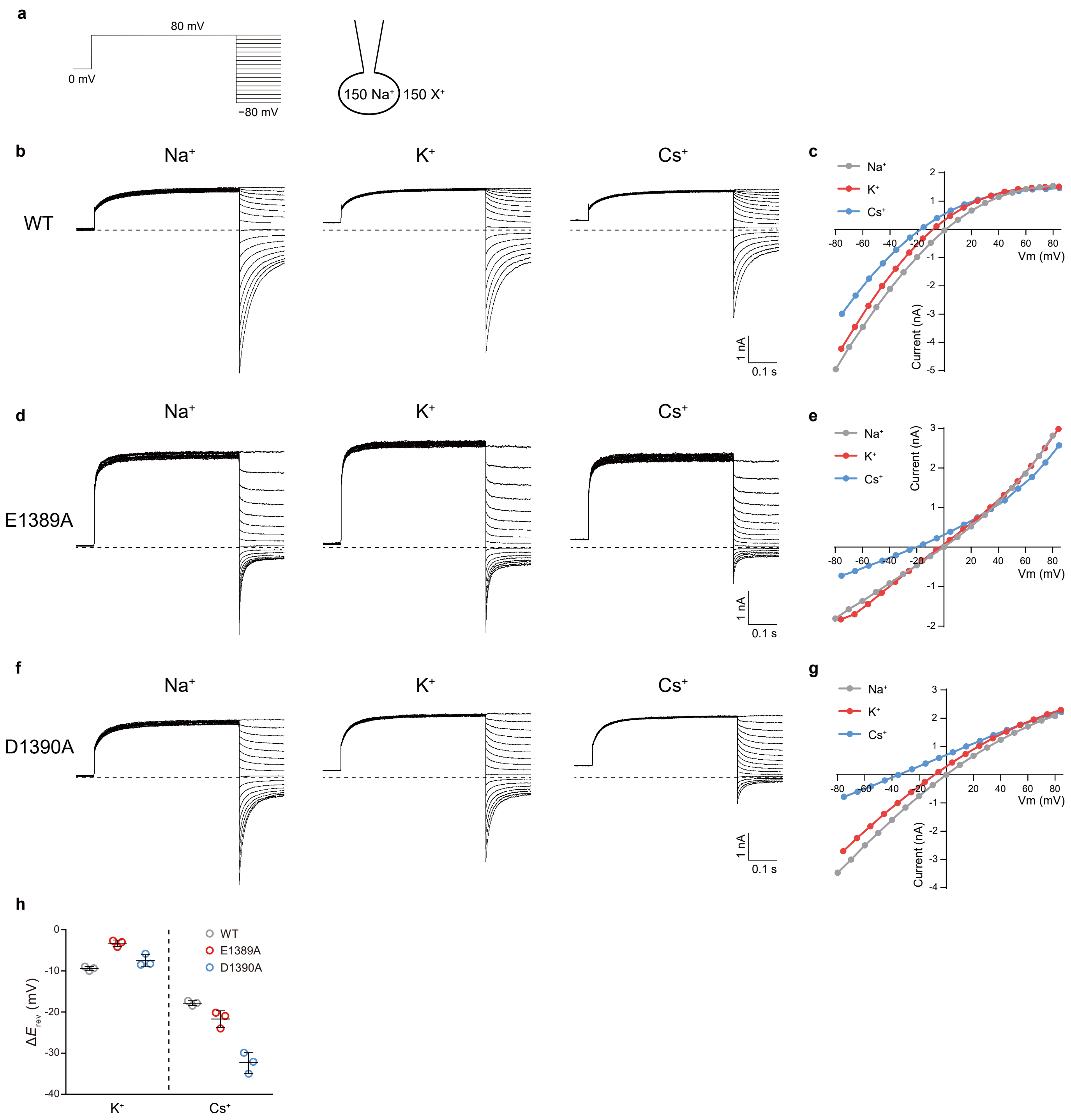

Supplementary Fig. 8 | Ion selectivity of NALCN and its mutants. a, The whole-cell step protocol used for reversal potential measurements with 150 mM Na^+ in the pipette solution and 150 mM X^+ (X = Na^+, K^+, Cs^+) in the bath solution. **b-g**, Representative NALCN current traces and corresponding I-V curves generated from tail currents of WT (**b-c**), E1389A mutant (**d-e**) and D1390A mutant (**f-g**) under various bi-ionic conditions. Dashed lines indicate the position of 0 nA. **h**, The reversal potentials of NALCN and its mutants ($\Delta E_{rev} = E_{rev}(X) - E_{rev}(Na)$, X = K^+, Cs^+) under bi-ionic conditions. Data are presented as mean ± s.d., n = 3 biologically independent cells. Source data are provided as a Source Data file.

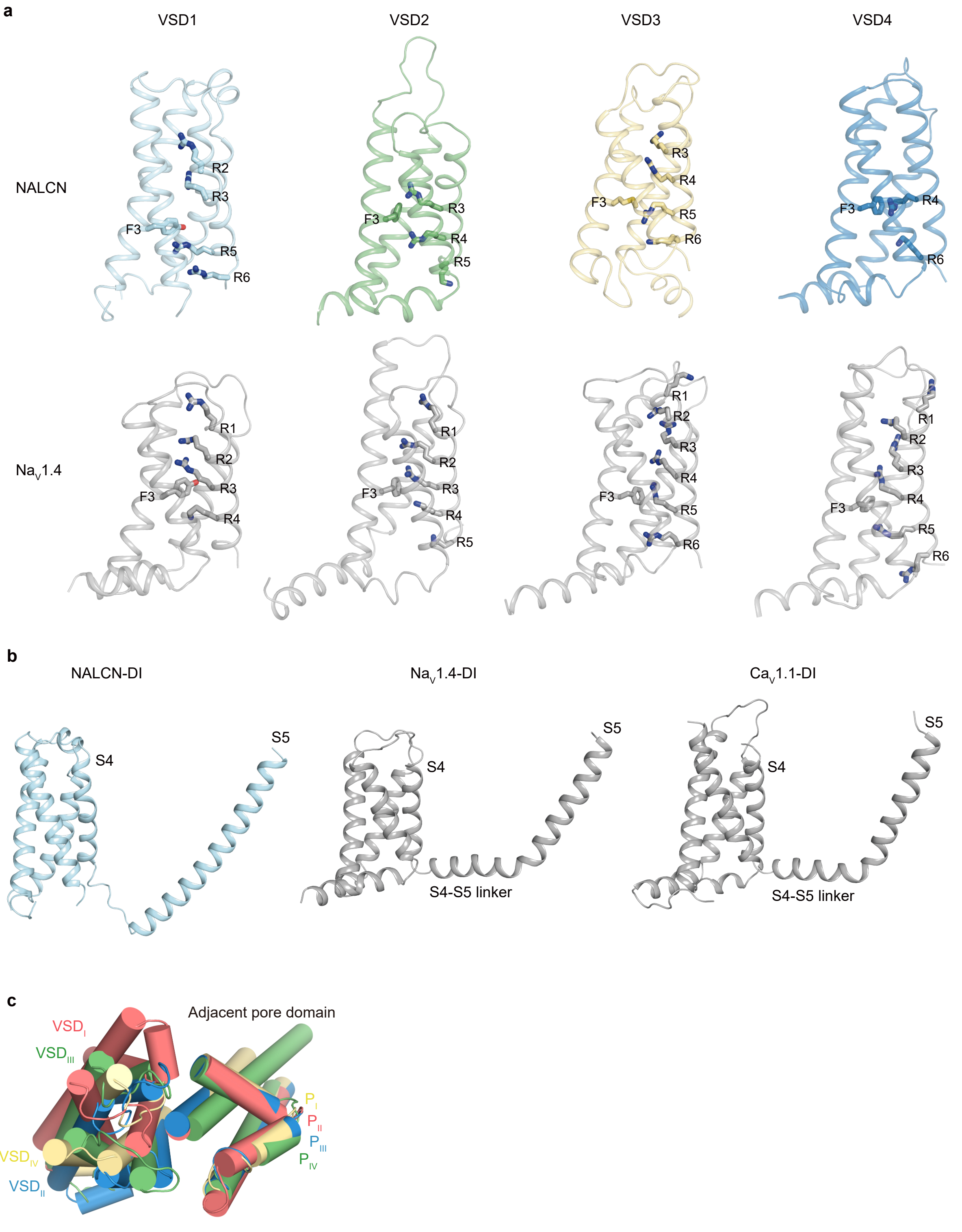

Supplementary Fig. 9 | Structure of voltage-sensor domains and S4-S5 linker of NALCN. **a**, Comparison of the VSDs from NALCN and Na$_V$1.4 (PDB ID: 6AGF). **b**, Comparison of the S4-S5 linker of DI from NALCN, Na$_V$1.4 (PDB ID: 6AGF) and Ca$_V$1.1 (PDB ID: 5GJV). Only S0-S5 segments of DI were shown for clarity. **c**, Structural comparison of VS and its adjacent pore domain of NALCN. Pore domains are aligned. Showing the position of DI-VS relative to the pore is different from others.

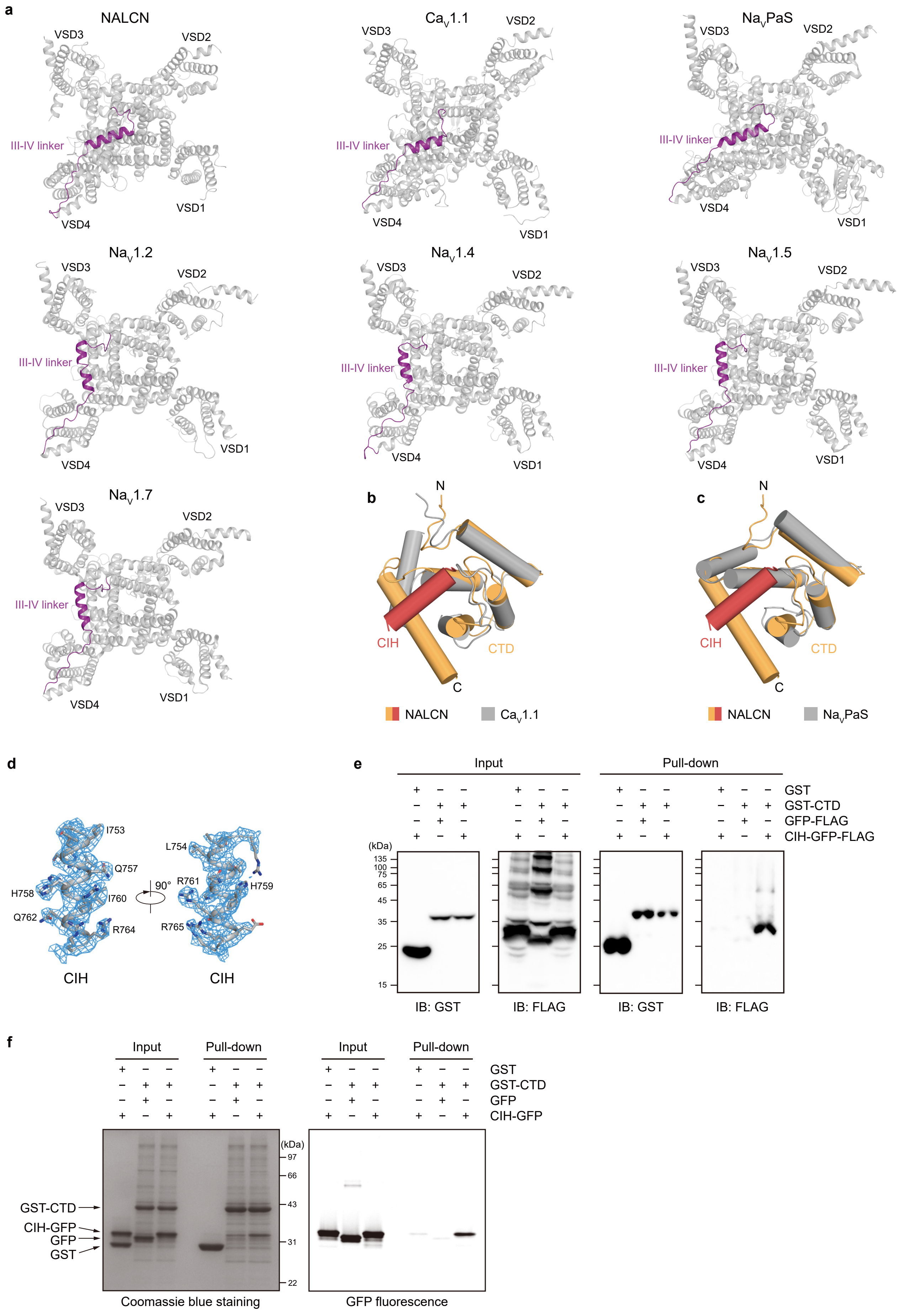

Supplementary Fig. 10 | NALCN III-IV linker and C-terminal domain comparisons. a, Comparison of III-IV linker (bottom view) from NALCN, Ca_V1.1 (PDB ID: 5GJV), Na_VPaS (PDB ID: 5X0M), Na_V1.2 (PDB ID: 6J8E), Na_V1.4 (PDB ID: 6AGF), Na_V1.5 (PDB ID: 6UZ3) and Na_V1.7 (PDB ID: 6J8G). III-IV linkers are colored by purple. **b-c**, Comparison of the CTD from NALCN, Ca_V1.1 (PDB ID: 5GJV) and Na_VPaS (PDB ID: 5X0M). CIH of NALCN is colored by red. Helices are shown as cylinders. **d**, Electron density of CTD Interacting Helix (CIH) on II-III linker of NALCN, is contoured at 3.5 σ. **e**, GST pull-down assay result showing interactions between NALCN CTD (residues 1455-1571) and CIH (residues 748-767) when co-transfected in HEK293F. The experiments were repeated three times with similar results. The original blots were provided as a Source Data file. **f**, GST pull-down assay result showing interactions between NALCN CTD and CIH which were both purified from *E.coli*. The experiments were repeated three times with similar results. The original gel was provided as a Source Data file.

Supplementary Table 1 | Cryo-EM data collection, refinement and validation statistics

	NALCN-FAM155A
PDB ID	7CU3
EMDB ID	EMD-30470
**Data collection and processing**	
Magnification	130,000 ×
Voltage (kV)	300
Electron exposure ($e^-/Å^2$)	50.9
Defocus range (μm)	-1.5 to -1.8
Pixel size (Å)	1.045
Symmetry imposed	*C1*
Final particle images (no.)	135,043
Map resolution (Å)	
Unmasked	3.4
Masked	2.7
FSC threshold	0.143
Map sharpening *B* factor ($Å^2$)	-78.6
**Refinement**	
Initial model	5GJV
Model composition	
Non-hydrogen atoms	12,209
B factors ($Å^2$)	
Protein	44.97
Ligand	39.47
R.m.s. deviations	
Bond lengths (Å)	0.006
Bond angles (°)	0.671
Validation	
MolProbity score	2.09
MolProbity percentile	71
Clashscore	6.59
Poor rotamers (%)	3.43
Ramachandran plot	
Favored (%)	95.39
Allowed (%)	4.61
Disallowed (%)	0.00
